# Larval outbreaks in West Greenland: Instant and subsequent effects on tundra ecosystem productivity and CO_2_ exchange

**DOI:** 10.1007/s13280-016-0863-9

**Published:** 2017-01-23

**Authors:** Magnus Lund, Katrine Raundrup, Andreas Westergaard-Nielsen, Efrén López-Blanco, Josephine Nymand, Peter Aastrup

**Affiliations:** 1 Department of Bioscience, Arctic Research Centre, Aarhus University, Frederiksborgvej 399, 4000 Roskilde, Denmark; 2 Greenland Institute of Natural Resources, Kivioq 2, P.O. Box 570, 3900 Nuuk, Greenland; 3 Department of Geosciences and Natural Resource Management, Center for Permafrost (CENPERM), University of Copenhagen, Oestervoldgade 10, 1350 Copenhagen, Denmark

**Keywords:** Arctic, Carbon, Disturbance, Ecosystem productivity, *Eurois occulta*, Insect outbreak

## Abstract

Insect outbreaks can have important consequences for tundra ecosystems. In this study, we synthesise available information on outbreaks of larvae of the noctuid moth *Eurois occulta* in Greenland. Based on an extensive dataset from a monitoring programme in Kobbefjord, West Greenland, we demonstrate effects of a larval outbreak in 2011 on vegetation productivity and CO_2_ exchange. We estimate a decreased carbon (C) sink strength in the order of 118–143 g C m^−2^, corresponding to 1210–1470 tonnes C at the Kobbefjord catchment scale. The decreased C sink was, however, counteracted the following years by increased primary production, probably facilitated by the larval outbreak increasing nutrient turnover rates. Furthermore, we demonstrate for the first time in tundra ecosystems, the potential for using remote sensing to detect and map insect outbreak events.

**Electronic supplementary material:**

The online version of this article (doi:10.1007/s13280-016-0863-9) contains supplementary material, which is available to authorized users.

## Introduction

Arctic tundra ecosystems cover ca. 8% of the global land area. Yet, the vast stocks of organic carbon (C) stored in their soils make them especially important in a climate change context (McGuire et al. 2012), since increasing temperatures may result in increased emissions of carbon dioxide (CO_2_) and methane (CH_4_). The occurrence of periodic disturbances, such as fires, pathogens and insect outbreaks, which to a varying temporal and spatial extent damage vegetation and affect C cycling, are also likely to change in the future (Callaghan et al. 2004; Post et al. 2009). There are, however, large gaps in our understanding of how extreme events affect ecosystem functioning and they are as such generally underrepresented in process-based ecosystem models (McGuire et al. 2012).

Insect outbreaks can have extensive consequences for ecosystem productivity and functioning in subarctic and arctic biomes (Callaghan et al. 2004; Post et al. 2009). The outbreaks may lead to local and regional canopy defoliation (Tenow and Nilssen 1990; Callaghan et al. 2004; Bjerke et al. 2014), decreased vegetation biomass (Pedersen and Post 2008; Post and Pedersen 2008), shifts in vegetation composition (Karlsen et al. 2013; Jepsen et al. 2013), decreased C uptake (Heliasz et al. 2011) as well as cascading impacts through other food web compartments (Jepsen et al. 2013). The prevalence and intensity of these disturbances are expected to increase with a warmer climate (Neuvonen et al. 1999; Callaghan et al. 2004; Chapin et al. 2004). The strong warming observed in northern high latitudes (Stocker et al. 2013) has been associated with a northward extension of outbreaks of moths and their leaf-defoliating larvae in northern Fennoscandia (Post et al. 2009; Jepsen et al. 2013), likely related to enhanced survival of overwintering eggs due to warmer winters (Callaghan et al. 2004).

There are several reports of outbreaks of the autumnal moth *Epirrita autumnata* and the winter moth *Operophtera brumata* from northern Fennoscandia, occurring at roughly decadal intervals (Tenow and Nilssen 1990; Callaghan et al. 2004; Heliasz et al. 2011; Jepsen et al. 2013; Karlsen et al. 2013). The larvae of these moth species not only defoliate forests of mountain birch *Betula pubescens*, but have also been found to feed on understorey vegetation including dwarf birch *Betula nana* and bilberry *Vaccinium myrtillus* (Karlsen et al. 2013). During an extensive outbreak in the lake Torneträsk catchment in subarctic Sweden in 2004, the mountain birch forest was a much smaller C sink during the growing season compared with a reference year, most likely due to lower gross primary production (Heliasz et al. 2011). Furthermore, changes in light conditions caused by defoliation and nutrient additions from larval faeces and carcasses (Karlsen et al. 2013) may alter the conditions for plant species not directly affected by defoliation.

In Greenland, outbreaks of larvae of the noctuid moth *Eurois occulta* have occasionally been reported (see "Background" section). During the 2004–2005 outbreak in Kangerlussuaq, West Greenland, the above ground biomass of all plant functional groups was reduced by up to 90% as a result of intense defoliation (Post and Pedersen 2008). However, little is known about the frequency, timing and extent of the outbreaks of *E. occulta* in Greenland. The purpose of this study is therefore to synthesize available knowledge on *E. occulta* outbreaks in Greenland and their effects on ecosystem functioning and productivity. We were fortunate to document an outbreak of *E. occulta* larvae in 2011 in Kobbefjord, West Greenland, where an extensive monitoring programme has been ongoing since 2008. We aim to quantify the effects of the larval outbreak on the ecosystem productivity by analyses of monitoring data on land–atmosphere exchange of CO_2_ and vegetation greenness derived from an automatic camera setup. We study the effects of the larval outbreak over a longer time period including three years following the outbreak, allowing for an investigation of how the tundra ecosystem responds to the larval attack in subsequent years. Furthermore, we use satellite imagery to investigate possible historical outbreaks in the Kobbefjord catchment.

## Background

**Fig. 1:**
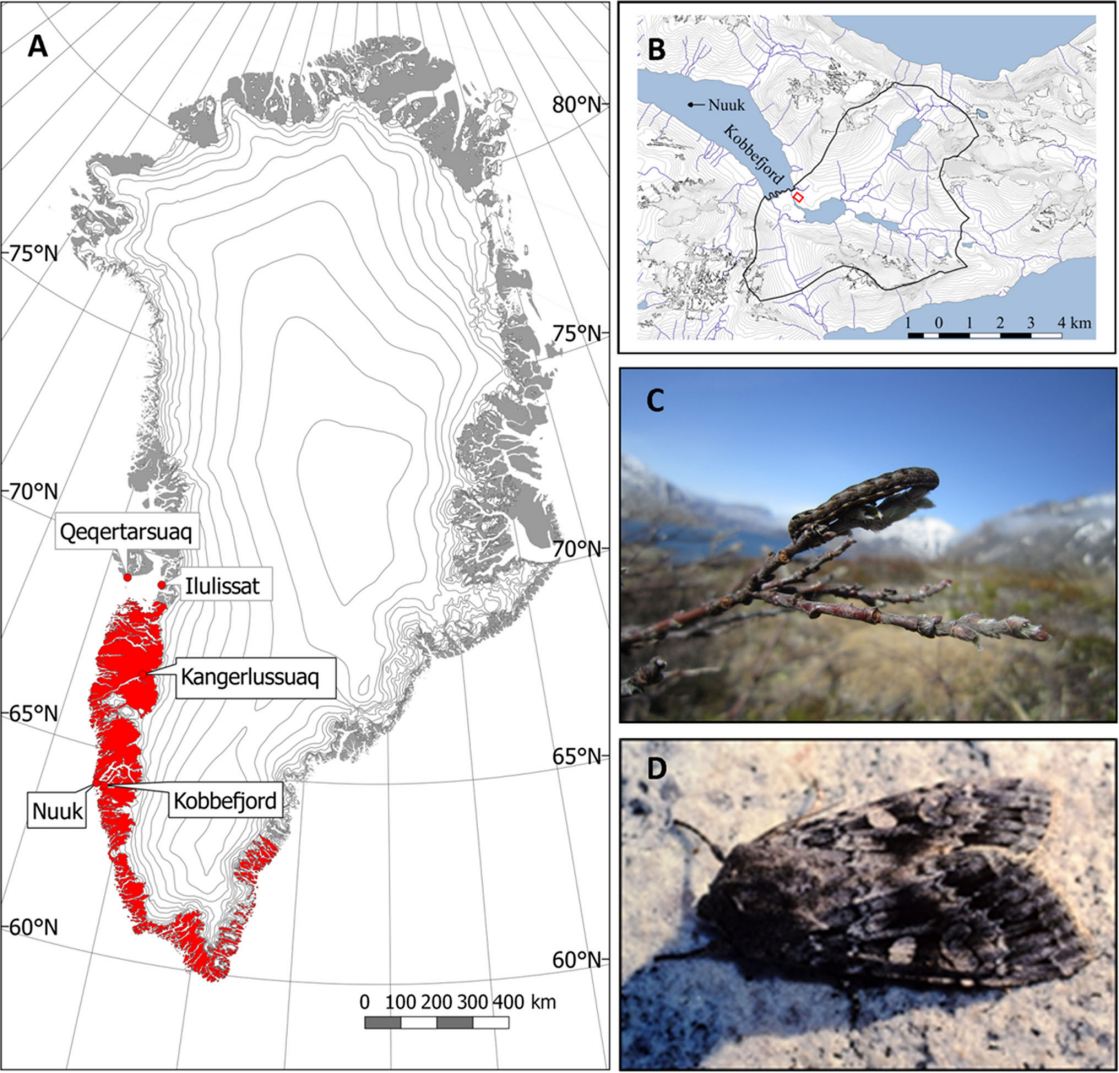
**a** Updated distribution of *Eurois occulta* in Greenland. The map is modified from Jensen (2003) and includes data points from Mølgaard et al. (2013). **b** Study area in Kobbefjord. The *red square* indicates the approximate location of the experimental plots. The *black line* delineates the watershed. **c** *E. occulta* larvae. **d** Adult *E. occulta* moth, photo: J. Böcher

*Eurois occulta* is a noctuid moth with a holarctic distribution. In Greenland, *E. occulta* is distributed northwards to Ilulissat and Qeqertarsuaq on the west coast (Mølgaard et al. 2013) and to Skjoldungen on the east coast (Fig. 1; Karsholt et al. 2015). Adult moths fly from early July to early September when they lay their eggs under stones or in moss. They hatch in fall and survive the winter as partially grown larvae underneath the snow before developing into fully grown larvae during the following spring. At this stage, they forage on green parts of the plants. In some years, *E. occulta* larvae occur in tremendous numbers (Vibe 1971).

These outbreak events have been reported to occur as far back as the late 1400 s as documented in peat cores from Ujarassuit (Iversen 1934). Since then, a number of *E. occulta* larvae outbreaks have been reported in Greenland (Table 1), i.e. in the Kangerlussuaq inland (Fox et al. 1987; Pedersen and Post 2008; Avery and Post 2013) and in the Nuup Kangerlua area (Iversen 1934), most recently in 2010–2011 when outbreaks occurred at both locations. *E. occulta* was also found in the Disko Bay region in 2012 (Mølgaard et al. 2013). Vibe (1971) reported that outbreaks of larvae have been observed often and must be regarded as a normal phenomenon. Although this has not been determined specifically for *E. occulta*, Vibe (1971) suggested that outbreaks in Greenland occur only under the right combinations of climatic factors, e.g. temperature, solar radiation, humidity, precipitation and wind.

**Table 1:** Reported outbreaks of *Eurois occulta* in Greenland

Year	Location	Latitude	Longitude	References
1490	Ameralik	64.22	−50.00	Iversen (1934)
1932	Ameralik	64.22	−50.00	Iversen (1934)
1932	Kangerlussuaq	67.03	−50.62	Iversen (1934)
1979	Kangerlussuaq	67.03	−50.62	Fox et al. (1987)
2004	Kangerlussuaq	67.03	−50.62	Pedersen and Post (2008)
2005	Kangerlussuaq	67.03	−50.62	Pedersen and Post (2008)
2010	Kangerlussuaq	67.03	−50.62	Avery and Post (2013)
2011	Kangerlussuaq	67.03	−50.62	Avery and Post (2013)
2011	Kobbefjord	64.13	−51.37	This study
2012	Ilulissat	69.25	−50.92	Mølgaard et al. (2013)
2012	Qeqertarsuaq	69.25	−53.55	Mølgaard et al. (2013)

## Materials and methods

### Study site

This study was conducted in Kobbefjord/Kangerluarsunnguaq in low Arctic West Greenland (64°08′N, 51°23′W, ca. 25 m a.s.l.), located ca. 20 km from Nuuk, the capital of Greenland (Fig. 1). This area is subjected to extensive monitoring and long-term research activities within the Greenland ecosystem monitoring (GEM) programme. The area is part of a valley system surrounded by mountains that reach up to ca. 1300 m a.s.l. The monitoring area covers 32 km^2^ and is characterised by dwarf shrub heaths intersected with dry south-facing slopes and smaller fen areas. The heaths are dominated by *Salix glauca*, *Betula nana* and *Empetrum nigrum* (Bay et al. 2008). Long-term (1961–1990) mean annual temperature and precipitation sum for Nuuk are −1.4 °C and 750 mm, respectively (Cappelen 2012).

### Monitoring data

Three terrestrial monitoring sub-programmes are operational in the Kobbefjord valley, namely BioBasis, GeoBasis and ClimateBasis (cf. Jensen and Rasch 2008); data from these programmes form the basis of this study. In 2008, an experiment was set up consisting of 18 control plots, six open-top ITEX chambers that increase temperature (cf. Henry and Molau 1997) and six plots with Hessian tents that reduce incoming light (Aastrup et al. 2015). In this study, only data from control plots were used.

Measurements of CO_2_ exchange were conducted weekly to biweekly during the snow-free season 2008–2014 using the closed chamber technique. A plexiglas measuring chamber (0.33 × 0.33 × 0.34 m), equipped with a fan for air mixing and a HTR-2 probe logging photosynthetic photon flux density and air temperature, was placed on top of a fixed metal frame for three minutes and air was analysed for CO_2_ concentrations using an infrared gas analyser EGM4 (PP Systems, USA). The linear change in CO_2_ concentration in the transparent chamber was used to calculate net ecosystem exchange (NEE), whereas a subsequent measurement in a dark chamber was used to represent ecosystem respiration (*R*_eco_). Gross primary production (GPP) was calculated as the difference between light and dark measurements (GPP = NEE − *R*_eco_).

All taxonomic groups of arthropods were sampled on a weekly basis at four sites located within a few hundred metres of the experimental plots, each with eight pitfall traps as specified by Aastrup et al. (2015). The traps contained ca. 200 ml water with one teaspoon of salt and two drops of detergent. The number of *E. occulta* larvae was counted at the department of Bioscience, Aarhus University, Denmark. For the purpose of this paper, samples from one site (arthropod plot 3) with vegetation composition and coverage resembling the CO_2_ flux plots were included in the analyses.

Soil temperatures (ST) from a depth of 1, 5, 10 and 30 cm were measured with T107 temperature probes (Campbell Sci., UK) approximately 500 m from the experimental plots. Incoming photosynthetic photon flux density (PPFD; Li-190SA, LICOR, USA) and air temperature (AT; Vaisala HMP 45D, Finland) were obtained from a weather station located ca. 2 km from the experimental plots. Daily imagery of the valley was derived from a HP E427 digital camera housed inside a weatherproof box. The box was mounted at 500 m above sea level in September 2009, and daily images were taken at noon local time (Westergaard-Nielsen et al. 2013).

### Data analyses

The CO_2_ flux measurements provide a snapshot of the CO_2_ exchange at the specific time of the day when measurements were performed. In order to take diurnal variation into account and to estimate seasonal budgets, the following nonlinear equations (Saarnio et al. 2003; Lund et al. 2009) were parameterised for each year separately based on available monitoring data:

1 $$ {\text{GPP}} = \frac{{a \times {\text{PPFD}} \times {\text{ST}}}}{{b + {\text{PPFD}}}} $$

2 $$ R_{\text{eco}} = c \times e^{{d\; \times \;{\text{ST}}}} $$

where *a*, *b*, *c* and *d* are regression parameters. Initial tests indicated that ST at 30 cm provided best fits (highest *r* ^2^ values) for GPP (Eq. 1), whereas ST at 5 cm was most suitable for *R*_eco_ (Eq. 2). The time series of GPP and *R*_eco_ were constructed between 1 June and 31 August for each plot and year, and NEE was calculated as the sum of GPP and *R*_eco_. The ST measurements were initiated on 25 July 2008 and thus, we did not estimate a seasonal budget for 2008.

**Fig. 2:**
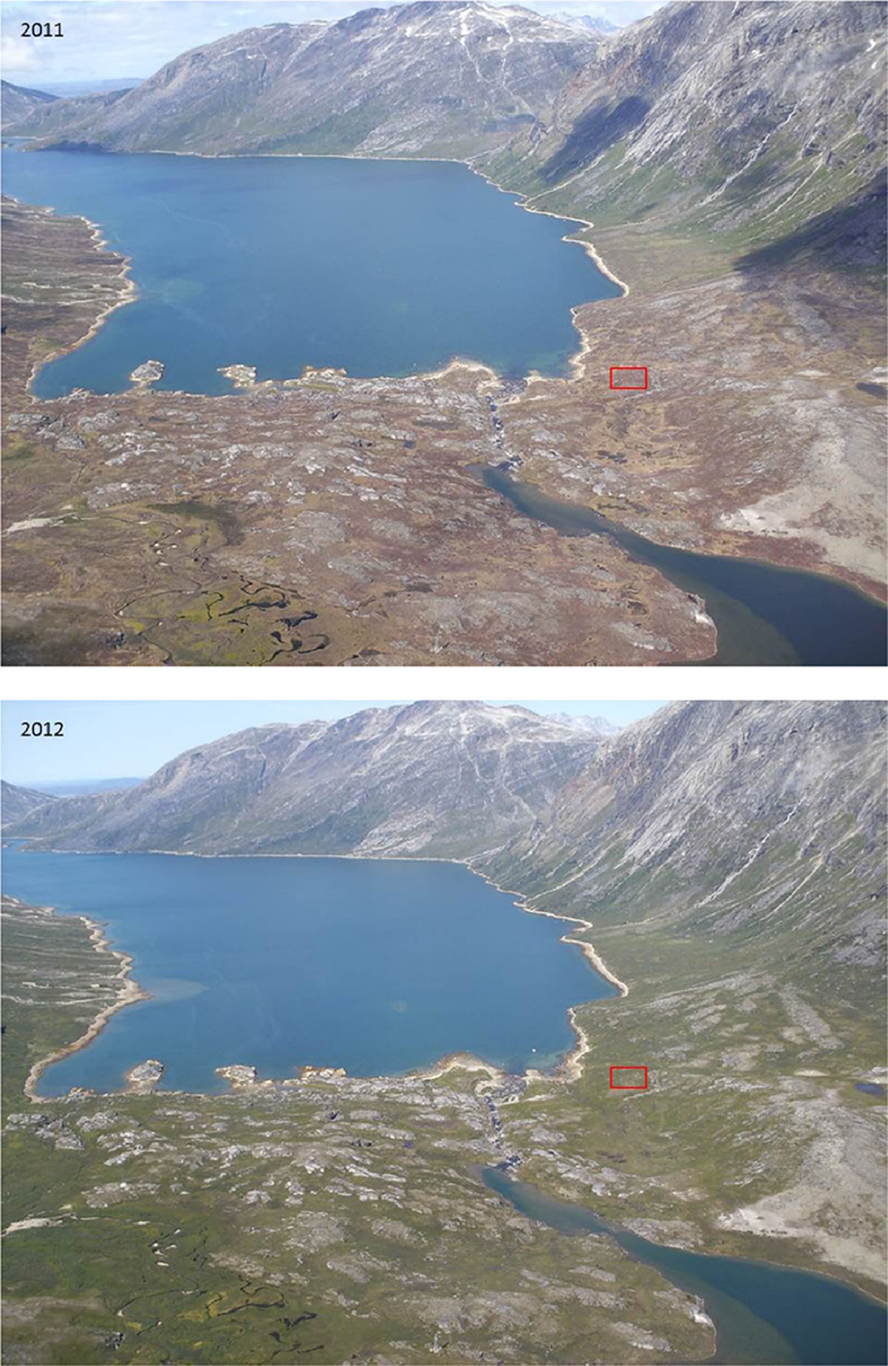
Photos from 20 July 2011 and 21 July 2012 from the fixed automatic camera in Kobbefjord. The *red square* indicates the approximate location of the experimental plots

The vegetation greenness at plant community level was evaluated from time series of RGB-images available from the fixed automatic camera overlooking Kobbefjord (Fig. 2). In this study, snow cover fraction and green chromatic coordinate (GCC) were computed for the region covering the experimental plots from daily images through the snow melt and snow-free period. GCC has been used as a proxy for NDVI and ecosystem productivity in different ecosystems (cf. Toomey et al. 2015) including low Arctic tundra and wetlands (Westergaard-Nielsen et al. 2013) and can be extracted from digital cameras offering only red, green and blue colour channels. To enable a quantification of larval impact, the area around the head of Kobbefjord, as defined by the field of view of the camera (Fig. 2), was classified. An image from peak growing season (July 29, 2011) was selected for maximum separation between affected and non-affected vegetated areas. Prior to the classification, the image was orthorectified (Corripio 2004). Since the classification was based on high spatial resolution data with only three colour channels (RGB), a nearest neighbour classification in eCognition Developer (Trimble, Inc.) was applied, based on a processing chain of (1) a multi-resolution segmentation; (2) a spectral difference segmentation; and (3) a supervised nearest neighbour classification.

### Satellite data

The MOD13Q1 vegetation index (VI) product was used to assess the spatial and temporal differences of VI signals at locations with reported larvae outbreaks. The VI product is derived from the MODIS sensor on board the Terra satellite platform. MOD13Q1 is a 16-day composite at 250 m spatial resolution based on cloud-free observations and includes, e.g. normalized difference vegetation index (NDVI) and enhanced vegetation index (EVI) measurements (Huete et al. 2002). The data were pre-processed based on the quality assessment layer, to include only observations with VI quality down to bit 1000, however, no further processing steps to adjust for possible differences in the number of observations within each composite were taken.

The impact of larval outbreaks was examined using a window of 3 × 3 satellite pixels, with the centre pixel covering the geographical coordinates of reported outbreaks (for Kobbefjord, we used the location of the experimental plots, whereas for Kangerlussuaq, we used the coordinates for site 1 in Young et al. 2016). Time-integrated NDVI and EVI, which have been found to correlate significantly with the aboveground phytomass in the Arctic (Westergaard-Nielsen et al. 2015), were calculated using five 16-day composites during DOY 177–241 in each year. Values below 0.2 and 0.1 for NDVI and EVI, respectively, were considered erroneous and replaced by linear interpolation. This was done for Kobbefjord NDVI and EVI on DOY 193, 2004; DOY 209, 2012 and DOY 241, 2013. No values were below the thresholds for Kangerlussuaq.

## Results

The study years (2008–2014) were generally warmer and wetter (Table 2) than the long-term mean (see “Study site” section). The year 2010 had the highest mean annual temperature (3.4°C), mainly due to unusually high temperatures in the winter months (mean temperature for January, February and December = −1.6°C). In the summer months (June–August), mean temperatures ranged between 8.3°C (2011) and 10.5°C (2012) for all years. Snow cover varied considerably in time and space during the study period. In 2010, there was only a thin snow pack that disappeared early, whereas in 2011, maximum daily snow depth reached 1.36 m and in the experimental CO_2_ flux plots, snow did not disappear until 12 June.

**Table 2:** Meteorological characteristics during the study period in Kobbefjord including snow characteristics (Max depth, m; DOY of melt in the CO_2_ flux plots, day of year), annual means (AT, air temperature, °C at 2 m; Precipitation, mm) and summer means from June, July and August (AT; Precip; PPFD, photosynthetic photon flux density, µmol m^−2^ s^−1^)

	Snow characteristics		Annual values		Summer (JJA) values		
Year	Max. Depth	DOY of melt	AT	Precip	AT	Precip	PPFD
2008	n/a	n/a	−0.7	1127	9.2	140	435
2009	0.91	154	−0.6	838	8.8	135	410
2010	0.33	118	3.4	905	10.4	314	364
2011	1.36	163	−1.6	n/a	8.3	193	356
2012	1.02	152	0.5	n/a	10.5	254	373
2013	0.53	149	0.2	1046	8.4	230	377
2014	1.1	153	−0.1	709	9.0	208	391

**Fig. 3:**
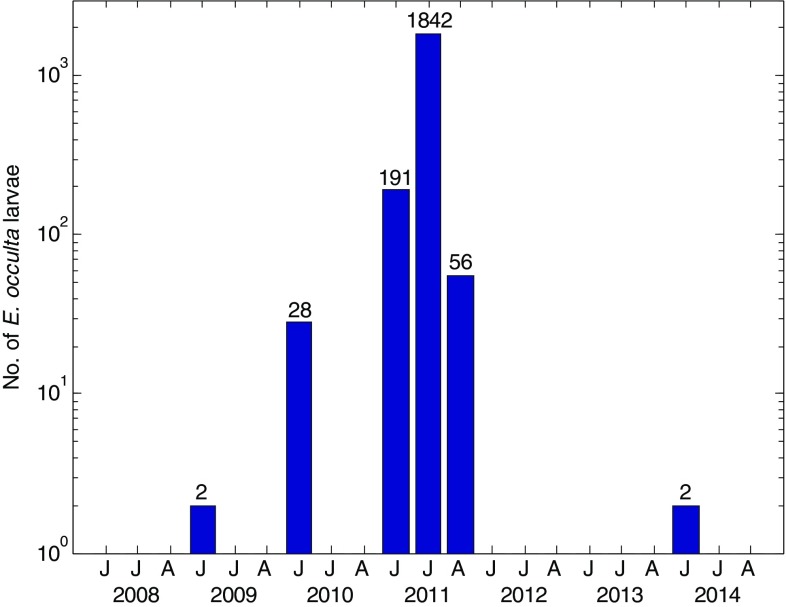
Number of *Eurois occulta* larvae in arthropod plot 3 in June, July and August (J, J, A) 2008–2014

**Fig. 4:**
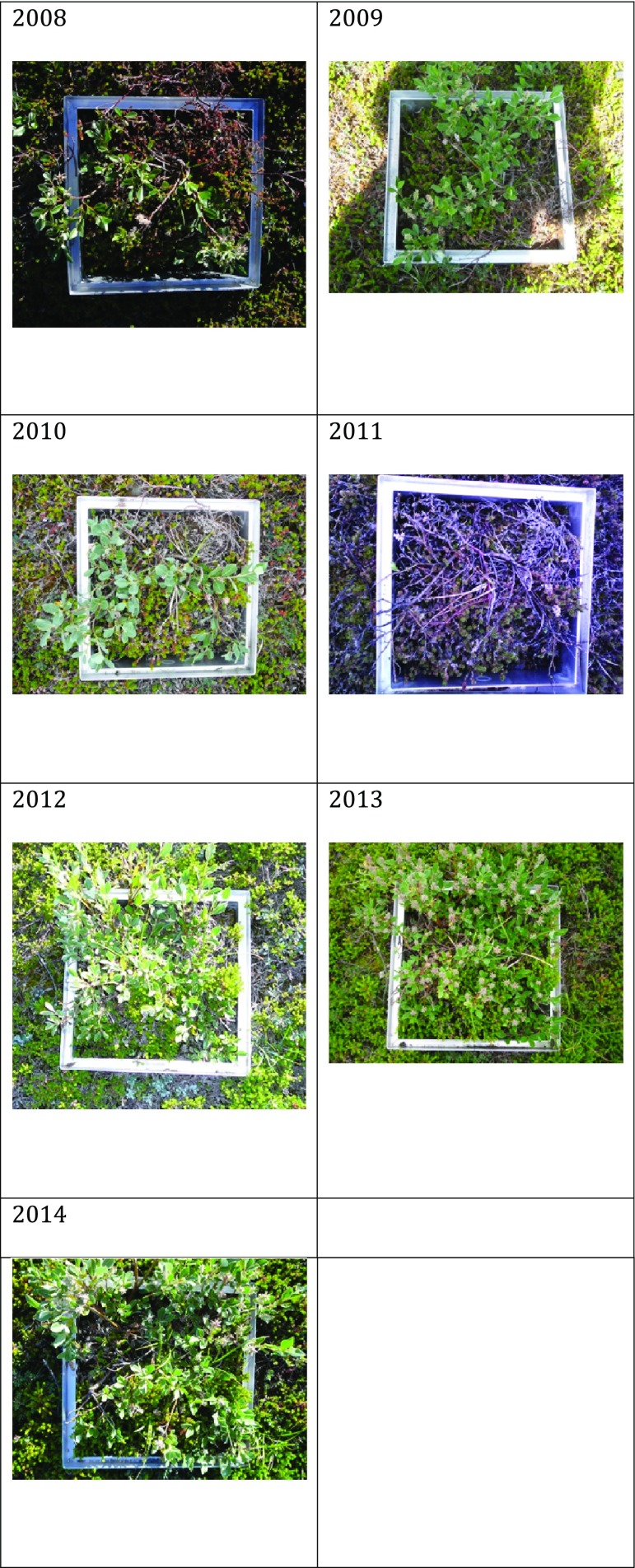
Yearly photos (2008–2014) from one of the experimental plots (plot 4C) in Kobbefjord. All photos were taken between DOY 203 and 208

Larvae of the noctuid moth *E. occulta* were observed in the Kobbefjord area mainly in 2010 and 2011. The number of larvae caught in pitfall traps in 2010 was modest with 31 larvae caught during the entire season. Numbers peaked in 2011 with more than 1800 larvae caught in July, thus representing an outbreak (Fig. 3). The dwarf shrub vegetation in the experimental CO_2_ flux plots was almost completely defoliated (Fig. 4). The amount of *Salix glauca* catkins was low in 2010 and non-existent in 2011, as a result of larvae feeding on the plants. Also, in 2012, when no larvae were encountered, no buds were developed. However, in the following years, the number of catkins was much higher compared with 2008–2012 (e.g. the *Salix* plants in plot 4 in Fig. 3 had 28, 21, 3, 0, 0, 143 and 73 female catkins in July 2008–2014, respectively).

**Fig. 5:**
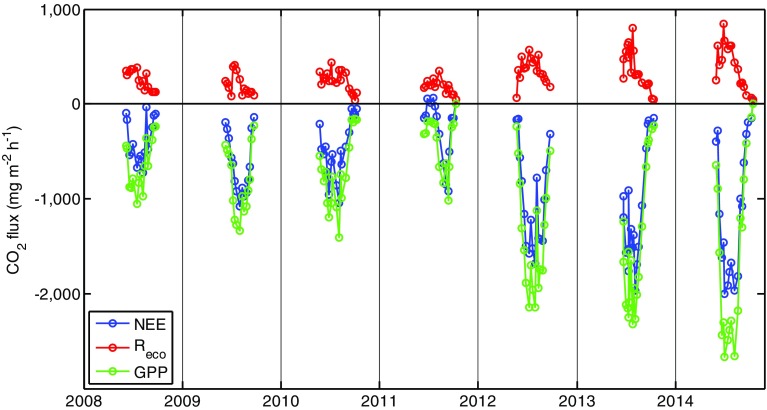
Instantaneous, daytime flux measurements of net ecosystem exchange (NEE) and ecosystem respiration (*R*_eco_) in the Kobbefjord experimental plots 2008–2014. Gross primary production (GPP) was calculated as the difference between NEE and *R*_eco_

The CO_2_ exchange measurements showed a distinct decrease in measured fluxes in the main outbreak year (2011) compared with the other years (Fig. 5), with lower amplitude of instantaneous NEE, *R*_eco_ and GPP. There was also an apparent delay in the onset of net CO_2_ uptake during 2011. However, the fluxes were higher in the years following the outbreak compared with earlier years. Based on the flux measurements and ancillary information on ST and PPFD, we parameterised Eqs. 1 and 2 separately for each year (2009–2014; Table S1). However, for the outbreak year of 2011, Eq. 1 could not be fitted with the data because the larvae damaged the vegetation and severely postponed the development of the photosynthetic apparatus, which in other years correlated with the progress in ST. As an alternative, we used the green chromatic coordinate (GCC) as a replacement for ST in Eq. 1, to be able to estimate a C budget in 2011 (Table S1).

As for the June–August budgets (Table 3), there were significant between-year differences in all flux components (ANOVA for GPP: *F* = 9.4, *p* < 0.001; *R*_eco_: F = 32.3, *p* < 0.001; NEE: *F* = 6.3, *p* < 0.001). Year 2011, the main larval outbreak year, had the lowest (i.e. least negative) GPP sums and a C sink close to zero (NEE = 7 ± 16 g C m^−2^). However, the years following the outbreak (2012–2014) had higher (i.e. more negative) GPP sums and stronger C sink strengths compared with the years preceding the outbreak (2009–2010).

**Table 3:** Mean ± standard error (spatial replication) of measured transparent (net ecosystem exchange, NEE) and dark (ecosystem respiration, *R*_eco_) fluxes and estimated budgets of gross primary production (GPP), *R*_eco_ and NEE during June–August 2008–2014 in Kobbefjord. Superscript letters for the budgets columns, derived from a Tukey’s HSD multiple comparison, indicate significant differences among years. Numbers in parentheses reflect sample size (i.e. number of plots)

Year	Measurements (mg CO_2_ m^−2^ h^−1^)		Budgets (g C m^−2^)		
	NEE	*R*_eco_	GPP	*R*_eco_	NEE
2008	−358 ± 81	262 ± 12	– ± –	– ± –	– ± –
2009	−497 ± 88	216 ± 13	−252 ± 39^abc^ (14)	100 ± 5^a^ (16)	−150 ± 40^abc^ (12)
2010	−496 ± 77	288 ± 14	−244 ± 21^ab^ (18)	150 ± 7^b^ (18)	−94 ± 17^ab^ (18)
2011	−86 ± 32	236 ± 9	−104 ± 14^a^ (10)	110 ± 3^a^ (17)	7 ± 16^a^ (10)
2012	−1023 ± 143	406 ± 21	−399 ± 49^bcd^ (13)	200 ± 10 ^cd^ (18)	−216 ± 44^bc^ (13)
2013	−1164 ± 178	425 ± 27	−424 ± 44 ^cd^ (18)	182 ± 10^bc^ (18)	−242 ± 37^bc^ (18)
2014	−1119 ± 160	479 ± 28	−505 ± 65^d^ (18)	231 ± 13^d^ (18)	−274 ± 54^c^ (18)

In order to estimate the C exchange in 2011, had there not been a larval outbreak, we used the parameterisations (Eqs. 1 and 2) from other years (2009, 2010, 2012–2014) with meteorological data (PPFD and ST) from 2011 (Table 4). The parameters in Eqs. 1 and 2 regulate the sensitivity of ecosystem photosynthesis and respiration to variations in PPFD and ST, and using meteorological data from 2011, we can assess the C budget assuming that the same sensitivity can be applied to 2011. Compared with the estimated flux component budgets in 2011 (Table 3), parameterisations from other years consistently provide higher (i.e. more negative) GPP sums and stronger C sink strengths (more negative NEE).

**Table 4:** Estimated flux components (GPP; gross primary production, *R*_eco_; ecosystem respiration, NEE; net ecosystem exchange, g C m^−2^) in 2011 using meteorological data (photosynthetic photon flux density, PPFD and soil temperature, ST) from 2011 and parameterisations of Eqs. 1 and 2 from other years (2009, 2010, 2012–2014)

Year	GPP	*R*_eco_	NEE
2009	−196 ± 31	84 ± 5	−111 ± 31
2010	−180 ± 16	119 ± 6	−61 ± 12
2012	−280 ± 34	158 ± 10	−136 ± 30
2013	−366 ± 37	170 ± 10	−196 ± 31
2014	−430 ± 57	205 ± 12	−226 ± 46

The green chromatic coordinate (GCC) for the area of the experimental plots (indicated by the red square in Fig. 2) was lower in 2011 compared with 2010 and 2012–2014 (Fig. S1). A time integration of GCC from the end of snow melt until the end of growing season suggests a 16% decrease in greenness in 2011 compared with the 2010–2014 average. The camera-based classification of the field of view of the camera in 2011 (Fig. 2) resulted in five separable ecosystem classes with an overall accuracy of 94% (Table S2); bedrock, non-attacked heath, larvae-attacked heath, fen and water (Table 5). The classification covered a total area of 2.58 km^2^, and we estimated that 0.83 km^2^ consisted of heath damaged by larvae; corresponding to 32% of the area covered by the camera (Fig. 2) or 59% of the total heath area.

**Table 5:** Area coverage from camera-based classification in Kobbefjord 2011

Class	Total area coverage (km^2^)	Fraction of classified area	Number of segments	Average area per segment (m^2^)
Bedrock	0.30	0.12	629	483
Non-attacked heath	0.57	0.22	298	1900
Attacked heath	0.83	0.32	694	1192
Fen	0.01	0.004	24	599
Water	0.87	0.34	89	9720

**Fig. 6:**
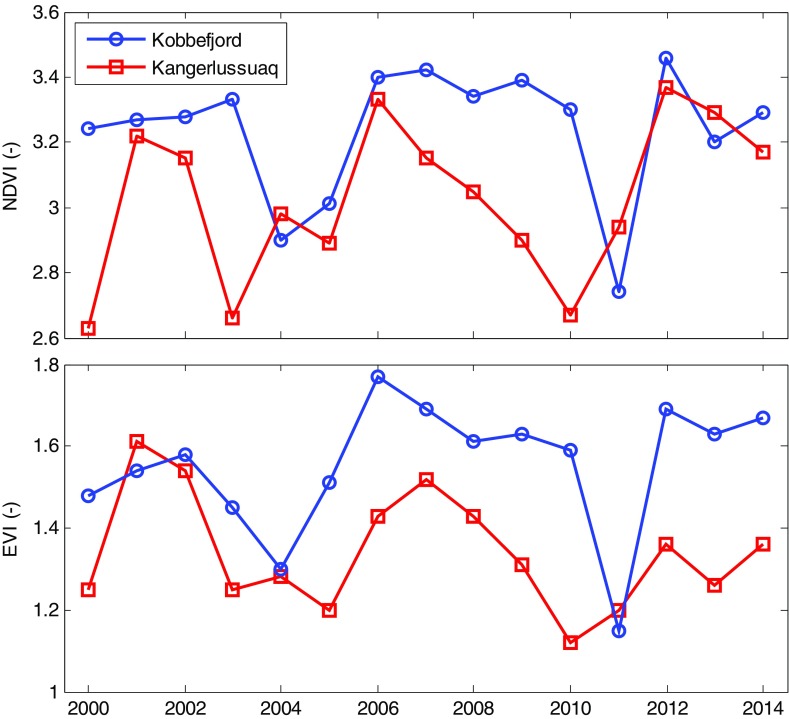
Time-integrated (DOY 177-241) normalized difference vegetation index (NDVI) and enhanced vegetation index (EVI) for Kobbefjord and Kangerlussuaq (Site 1 in Young et al. 2016), respectively, derived from the MODIS sensor

Time-integrated NDVI and EVI showed a marked decrease in 2011 in Kobbefjord (Fig. 6). Local minima were also observed in 2004 and 2005. Similarly, there were low values of NDVI and EVI for the area close to Kangerlussuaq for years with reported outbreaks, 2004–2005 and 2010–2011 (Table 1); however, the between-year variation was higher for this site with low time-integrated EVI and NDVI also in 2000 and 2003.

## Discussion

The outbreak of the noctuid moth *E. occulta* in Kobbefjord in 2011 had a strong and extensive impact on the vegetation. The production of leaves, buds and catkins or flowers in all species monitored in the area was heavily impacted and the vegetation reproduction was seriously reduced (Bay et al. 2012). Also, in the year following the outbreak, 2012, the total catkin and flower production was low (Bay et al. 2013), indicating that the plants focused their resources on establishing new leaves; a compensatory growth as a response to herbivory (McNaughton 1983). The excess energy stored in 2012 from not producing catkins resulted in a record amount of catkins in 2013 (e.g. Fig. 4).

The larvae did not forage upon leaves from all plant species, e.g. *Empetrum nigrum* was generally left untouched, although larvae feeding on *E. nigrum* flowers were observed in the field. *E. nigrum* is generally unpalatable to herbivores and not directly defoliated during moth outbreaks (Jepsen et al. 2013; Karlsen et al. 2013); however, previous studies have discussed the possibility that starving larvae attempt to eat their leaves making them more susceptible for desiccation or infection (Jepsen et al. 2013; Karlsen et al. 2013; Olofsson et al. 2013). Plot-scale NDVI measurements showed that *E. nigrum* was notably less green in 2011 than in other years (Olsen et al. 2014), which could thus be explained by the larval outbreak. However, other adverse effects such as frost damage (Bjerke et al. 2014) during early winter 2010/2011 cannot be excluded.

In the following years, *E. nigrum* was greener than in previous years, also before the outbreak (Olsen et al. 2014). This pertained to all plant species as seen by generally higher NDVI values measured after the outbreak; an indication of good health, which may stem from higher levels of nutrients made available for the plants from decomposed larvae. Arctic tundra vegetation is generally nutrient limited (cf. Chapin and Shaver 1985) and larvae faeces and carcasses can provide a nutrient pulse to the system (Kagata and Ohgushi 2012). Also, reduced plant nutrient uptake during 2011, as a consequence of reduced growth, may have resulted in excess nutrient availability in the following years. Post and Pedersen (2008) report a fourfold increase in nitrogen (N) concentration in leaf tissues of *S. glauca* and *B. nana* at the peak of the larval outbreak in Kangerlussuaq (Table 1) along with a rapid biomass recovery following the outbreak.

There was a marked decrease in CO_2_ fluxes in 2011, both in terms of instantaneous, measured fluxes (Fig. 5) and estimated summertime budgets (Table 3). Net ecosystem exchange was close to zero during June–August in 2011 (Table 3), indicating that the heath ecosystem, represented by the experimental plots, did not accumulate C during this period. The reduction in CO_2_ accumulation during 2011 was caused by a significant decrease in GPP to less than half of that in other years (Table 3). However, fluxes were higher in the years following the outbreak, again indicating a rapid ecosystem recovery after the larvae attack. The increase in GPP in 2012–2014 may be explained by an increase in nutrient availability due to the larval outbreak, as discussed above, favouring vegetation growth in subsequent years.

The rapid regrowth and the increase in primary productivity indicate that the tundra ecosystem may not be as vulnerable as anticipated with respect to these outbreaks. The ecosystem may have developed a high degree of resiliency as a response to outbreak events occurring at certain intervals. Our findings, that the years following the outbreak (2012–2014) had higher GPP and stronger C sink strengths compared with the years preceding the outbreak (2009–2010), correspond to the strong biomass recovery observed in Kangerlussuaq following a larval outbreak (Post and Pedersen 2008). This indicates that the effects of outbreaks may be counterbalanced by increased primary production in the following years. Further, the larvae appear to play a significant role by influencing nutrient dynamics and accelerating N turnover.

By using the parameterisations from other years, we estimated what the CO_2_ exchange in 2011 would have been in the absence of the larval outbreak (Table 4). This approach takes inter-annual variation in meteorological characteristics into account, e.g. the impact of long-lasting snow cover in 2011 is included in these estimates. Parameters from all other years resulted in higher (i.e. more negative) GPP and stronger C sink strength, whereas the effect on *R*_eco_ was less consistent. The parameters from 2010 resulted in lower (less negative) GPP sum and weaker C sink strength compared with other years, which can be associated with the modest number of moth larvae affecting ecosystem productivity also in 2010 (Fig. 3). The parameters from 2013 and 2014 resulted in high GPP sums and strong C sink strengths, indicating a strong recovery from the larval attack and a potential switch to a more productive state as discussed above. As such, when assessing the effect of the larvae on vegetation productivity and C budget in 2011, it seems most reasonable to use parameters from 2009 and 2012. Although a simplification, it results in a decrease in C sink strength in the order of 118 to 143 g C m^−2^, with an associated uncertainty (combined standard error from the spatial replication) of approximately ± 47 g C m^−2^. Scaling to the field of view of the camera (Fig. 2), taking the whole area of attacked heath into account (Table 5), results in a C loss of 98–119 tonnes C. However, the camera covers <10% of the entire Kobbefjord catchment (2.58 out of 32 km^2^) so the catchment scale C loss may be one order of magnitude larger (approximately 1210–1470 tonnes C). This approximation can be compared with a study in northern Sweden by Heliasz et al. (2011), who estimated a C loss of 29 000 tonnes C for a mountain birch forest area of 316 km^2^ exposed to an outbreak of larvae in 2004 of the autumnal moth *E. autumnata*.

We demonstrate the potential for using satellite imagery to detect and map insect outbreaks in West Greenland. To our knowledge, this is the first time satellite data have been used to observe effects of insect outbreaks in tundra ecosystems. Both NDVI and EVI show a clear decrease in time-integrated values during 2011 in Kobbefjord, with a decrease of 15 and 26%, respectively, compared with the 2000–2014 mean (Fig. 6). A decrease of 16% in time-integrated GCC in 2011 matches this range. These estimates also match a 16–27% decrease in peak NDVI in 2012 in northern Fennoscandia during a moth larvae outbreak, compared with a 2000–2011 average (Bjerke et al. 2014). The outbreaks in Kangerlussuaq in 2004–2005 and 2010–2011 (Pedersen and Post 2008; Avery and Post 2013; Table 1) are also visible through low values of time-integrated indices (Fig. 6); however, the picture is less clear for this site with low values also in other years.

It might be suggested that outbreaks of *E. occulta* in West Greenland occur in synchrony since the satellite data indicate low NDVI and EVI in Kobbefjord also in 2004; a year with documented outbreak in Kangerlussuaq (Pedersen and Post 2008). However, as the current study is limited to the near vicinity of field investigations in the respective site, impacts on a larger scale can only be speculated upon. Nevertheless, spatial synchrony in outbreak events indicates that climatic variations play a key role in triggering outbreak events (Klemola et al. 2006; Young et al. 2014). Outbreaks of *Epirrita autumnata* and *Operophtera brumata* in northern Fennoscandia have been associated with reduced egg mortality during warm winters (Tenow and Nilssen 1990; Callaghan et al. 2004; Chapin et al. 2004; Young et al. 2014) as well as with decreased parasitoid and predator activity because of low spring and summer temperatures (Virtanen and Neuvonen 1999; Callaghan et al. 2004). However, other studies have found a positive relationship between moth outbreaks and spring and summer temperatures (Klemola et al. 2003; Young et al. 2014). In our study in Kobbefjord, the winters (December–February) of 2009/2010 and 2010/2011 were indeed the warmest on record with mean temperatures of −2.7 and −4.5 °C, respectively, compared with a 2008–2014 mean of −6.7 °C (Table 2). Also, the summer of 2011 was relatively cold. It is also worth mentioning the importance of snow, which plays a key role in regulating Arctic ecosystem functioning (cf. Callaghan et al. 2012). There was a thick and long-lasting snow pack in the winter 2010/2011 in Kobbefjord (Table 2), which insulated and protected overwintering *E. occulta* larvae from low winter temperatures. In line with this argumentation, it can be noticed that heath vegetation on mountain slopes does not appear to be affected by the larvae (Fig. 2); these areas are generally colder and covered by less snow than the lowlands.

## Conclusions

Our results indicate a marked decline in summertime C uptake during an outbreak of the larvae of *E. occulta* in 2011 in Kobbefjord. However, the years following the outbreak (2012–2014) were characterised by stronger C uptake compared with the years preceding the outbreak. This indicates that the ecosystem is well adapted to these outbreaks and that they presumably occur at certain intervals if a number of environmental conditions are fulfilled. As a consequence of the outbreaks, nutrient turnover rates increase and growth is favoured in subsequent years. As such, the outbreaks may facilitate ecosystem rejuvenation (Tenow et al. 2004).

Future studies should focus on developing tools based on remote sensing products such as the vegetation indices used here for mapping larval outbreak events in West Greenland. A spatially distributed dataset of outbreak events, as opposed to occasional observations, would be highly useful for comparisons with gridded climate data. However, satellite data can only provide landscape scale information on net effects. There is thus an urgent need to continue and expand upon in situ environmental monitoring efforts in the Arctic, in order to improve upon the process-based understanding of how climate change and associated changes in extreme events such as insect outbreaks may affect tundra ecosystem functioning and dynamics. Predicting extreme events, e.g. larval outbreaks, is difficult so in order to capture the events continuous, long-term monitoring programmes are required.

## Electronic supplementary material

Below is the link to the electronic supplementary material.

Supplementary material 1 (PDF 503 kb)
