# Supplementary material for: Larval outbreaks in West Greenland: Instant and subsequent effects on tundra ecosystem productivity and CO2 exchange
Source: Ambio. 2017 Jan 23;46(Suppl 1):26–38. doi: 10.1007/s13280-016-0863-9 (PMC5258657; doi:10.1007/s13280-016-0863-9)
Supplement: Supplementary file 1 — Supplementary material 1 (PDF 503 kb) [file 13280_2016_863_MOESM1_ESM.pdf]

Ambio

Electronic Supplementary Materials
This supplementary material has not been peer reviewed.

Title: **Larval outbreaks in West Greenland: instant and subsequent effects on tundra ecosystem productivity and CO_2 exchange**

Authors: Magnus Lund, Katrine Raundrup, Andreas Westergaard-Nielsen, Efrén López Blanco, Josephine Nymand, Peter Aastrup

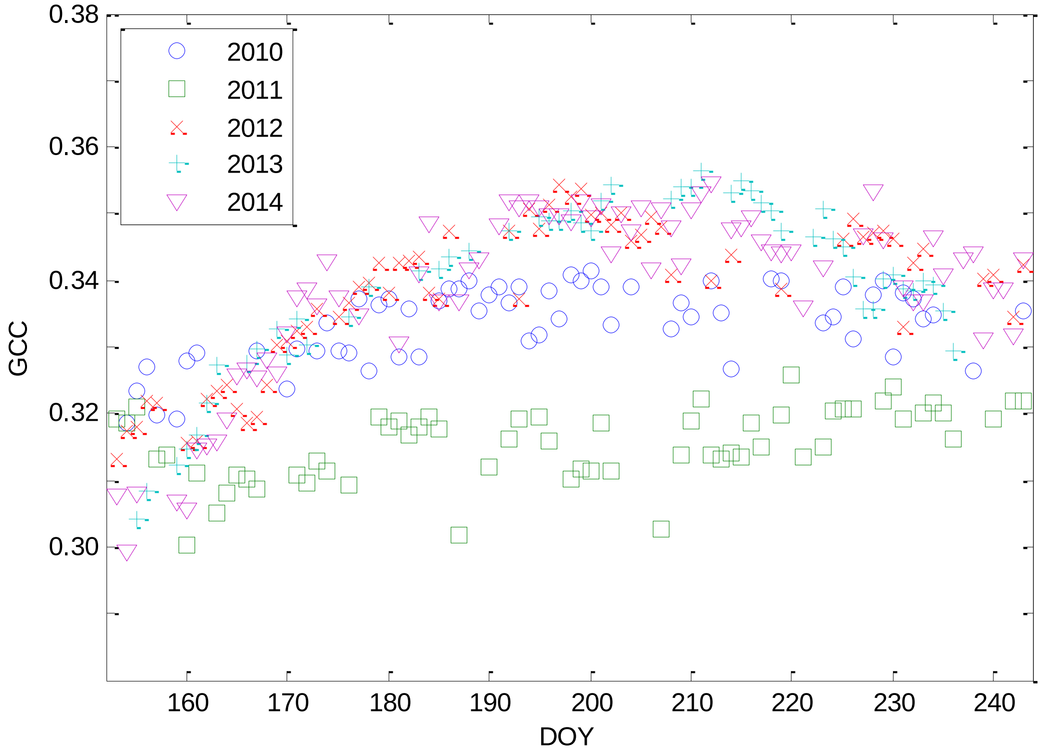

Fig. S1. The green chromatic coordinate (GCC) during June-August (DOY 152-243) in 2010-2014 for the area of the experimental plots (indicated by the red square in Fig. 2), derived from the fixed automatic camera.

Table S1. Estimated parameters from eq. 1 and eq. 2 used to construct time series of gross primary production (GPP) and ecosystem respiration (R_{eco}) for the experimental plots in Kobbefjord. Note that in 2011, the green chromatic coordinate (GCC) were used instead of soil temperature (ST) in eq. 1.

Year	Plot no.	GPP parameterization (eq. 1)					R_{eco} parameterization (eq. 2)				
		a	b	r2	RMSE	n	c	d	r2	RMSE	n
2009	1	-145	310	0.86	146	15	65	0.10	0.53	55	15
	2	-183	192	0.76	287	14	54	0.11	0.40	75	14
	3	-63	260	0.31	123	13	17	0.20	0.66	61	13
	4	-	-	-	-	-	7	0.30	0.93	65	13
	5	-	-	-	-	-	53	0.12	0.53	76	14
	6	-143	33	0.59	371	14	53	0.14	0.54	108	14
	7	-62	391	0.69	83	14	30	0.13	0.49	37	14
	8	-211	509	0.76	304	14	-	-	-	-	-
	9	-52	89	0.46	123	14	14	0.22	0.71	67	14
	10	-62	47	0.32	146	13	23	0.19	0.60	91	13
	11	-122	199	0.54	360	13	40	0.16	0.79	66	13
	12	-	-	-	-	-	28	0.16	0.62	67	14
	13	-36	168	0.72	55	14	12	0.21	0.69	42	14
	14	-202	134	0.63	392	15	74	0.10	0.39	85	15
	15	-47	215	0.40	99	14	45	0.14	0.47	92	14
	16	-65	85	0.47	149	14	19	0.20	0.63	92	14
	17	-56	132	0.33	149	14	-	-	-	-	-
	18	-	-	-	-	-	27	0.16	0.69	55	14
2010	1	-123	200	0.63	228	17	72	0.11	0.66	57	18
	2	-189	533	0.45	460	17	84	0.10	0.42	89	17
	3	-66	196	0.71	99	17	41	0.14	0.49	69	17
	4	-140	198	0.78	259	17	48	0.17	0.81	76	17
	5	-174	369	0.76	278	15	66	0.13	0.63	86	15
	6	-93	178	0.86	101	17	74	0.11	0.64	67	17
	7	-46	88	0.78	64	18	35	0.14	0.85	26	18
	8	-203	588	0.83	239	16	79	0.09	0.40	76	18
	9	-90	220	0.80	83	17	63	0.13	0.66	77	18
	10	-81	116	0.71	142	17	50	0.15	0.79	58	17
	11	-84	198	0.88	73	15	65	0.11	0.63	65	15
	12	-145	161	0.76	273	17	152	0.08	0.41	131	17
	13	-49	139	0.76	81	17	64	0.11	0.77	36	18
	14	-244	682	0.64	424	18	79	0.12	0.43	115	18
	15	-73	163	0.39	274	16	61	0.14	0.69	74	18
	16	-76	125	0.77	101	17	46	0.14	0.76	53	17
	17	-38	94	0.34	123	13	34	0.16	0.67	70	15
	18	-83	617	0.82	92	17	109	0.06	0.34	72	17
2011	1	-84	67	0.66	114	12	66	0.12	0.74	45	14
	2	-	-	-	-	-	68	0.09	0.43	49	12

	3	-97	647	0.54	96	13	89	0.08	0.58	43	14
	4	-122	640	0.22	263	10	64	0.12	0.71	64	11
	5	-	-	-	-	-	121	0.06	0.41	80	10
	6	-	-	-	-	-	106	0.07	0.42	65	11
	7	-	-	-	-	-	41	0.13	0.64	33	12
	8	-	-	-	-	-	36	0.18	0.65	62	12
	9	-130	693	0.36	107	14	55	0.14	0.83	42	14
	10	-61	75	0.37	97	11	65	0.11	0.71	46	12
	11	-	-	-	-	-	108	0.07	0.58	56	11
	12	-	-	-	-	-	-	-	-	-	-
	13	-30	142	0.18	43	13	61	0.12	0.76	36	14
	14	-110	46	0.53	419	11	26	0.19	0.58	71	12
	15	-147	1369	0.80	57	13	47	0.15	0.95	22	14
	16	-175	775	0.69	60	13	27	0.19	0.92	32	14
	17	-	-	-	-	-	93	0.09	0.64	64	12
	18	-133	1571	0.31	147	11	132	0.05	0.51	46	12
2012	1	-193	368	0.86	197	17	84	0.12	0.57	104	17
	2	-239	245	0.87	282	17	152	0.06	0.25	131	17
	3	-113	129	0.75	127	16	97	0.10	0.77	59	16
	4	-	-	-	-	-	200	0.07	0.52	107	15
	5	-	-	-	-	-	95	0.12	0.45	106	12
	6	-	-	-	-	-	102	0.11	0.75	72	15
	7	-83	244	0.70	138	16	103	0.06	0.27	80	16
	8	-333	257	0.84	472	17	122	0.09	0.60	91	17
	9	-113	105	0.63	147	15	126	0.09	0.42	117	15
	10	-139	318	0.26	328	15	119	0.09	0.38	127	15
	11	-153	162	0.72	262	11	135	0.08	0.26	88	11
	12	-	-	-	-	-	257	0.06	0.51	99	14
	13	-73	71	0.71	142	16	148	0.05	0.32	71	17
	14	-276	294	0.89	294	14	130	0.09	0.53	104	14
	15	-	-	-	-	-	94	0.12	0.45	131	14
	16	-118	111	0.65	163	15	57	0.14	0.56	105	15
	17	-90	145	0.71	226	11	13	0.25	0.61	134	12
	18	-103	153	0.48	217	14	116	0.08	0.39	71	14
2013	1	-188	175	0.82	230	16	65	0.14	0.70	77	16
	2	-481	758	0.94	299	15	31	0.23	0.66	158	16
	3	-143	64	0.77	124	15	68	0.15	0.76	89	15
	4	-374	369	0.81	478	16	103	0.15	0.61	200	16
	5	-390	325	0.83	470	14	162	0.11	0.52	203	15
	6	-227	281	0.91	191	16	46	0.19	0.70	137	16
	7	-95	321	0.84	104	15	47	0.14	0.82	37	16
	8	-453	374	0.89	499	14	109	0.10	0.65	89	15
	9	-172	328	0.75	142	15	81	0.15	0.76	102	15
	10	-146	158	0.81	134	15	93	0.13	0.57	138	15

	11	-264	415	0.92	214	15	67	0.15	0.67	136	15
	12	-397	390	0.87	410	15	117	0.13	0.70	142	16
	13	-86	163	0.67	131	15	96	0.09	0.55	68	16
	14	-427	736	0.88	409	15	47	0.19	0.82	87	15
	15	-95	7	0.71	148	16	54	0.18	0.81	94	16
	16	-164	317	0.79	156	15	52	0.18	0.52	166	15
	17	-121	323	0.86	137	15	40	0.16	0.73	84	15
	18	-147	404	0.84	153	15	87	0.11	0.42	136	15
2014	1	-223	247	0.67	356	15	163	0.08	0.27	142	15
	2	-331	186	0.64	731	16	174	0.08	0.42	155	16
	3	-159	201	0.46	343	13	111	0.12	0.69	129	13
	4	-340	145	0.57	743	12	116	0.14	0.52	252	12
	5	-371	52	0.70	565	12	247	0.09	0.38	285	12
	6	-237	122	0.82	314	13	95	0.12	0.78	94	13
	7	-114	306	0.83	123	16	75	0.12	0.78	50	16
	8	-506	446	0.75	779	15	101	0.13	0.59	134	15
	9	-144	293	0.33	351	16	93	0.13	0.59	148	16
	10	-152	226	0.61	237	12	99	0.13	0.42	251	13
	11	-231	224	0.87	225	13	99	0.13	0.70	140	13
	12	-349	48	0.71	632	12	274	0.07	0.36	186	12
	13	-112	427	0.75	139	15	95	0.10	0.56	80	16
	14	-319	318	0.73	501	15	178	0.08	0.31	169	16
	15	-120	303	0.45	175	15	100	0.12	0.89	61	15
	16	-136	120	0.59	203	13	82	0.13	0.87	59	13
	17	-111	163	0.70	191	13	107	0.10	0.48	139	13
	18	-144	53	0.66	199	11	166	0.08	0.42	123	11

Table S2. Error matrix from the ecosystem classification. The classification is the result of an automated multiresolution segmentation process (eCognition Developer 9.0.2, Trimble) with primary weight on segment size (scale parameter set to 500) as well as shape (set to 0.1) and compactness (set to 0.5). The generated segments were subsequently used as training data (15 segments per class). The classification was based on a supervised maximum likelihood algorithm, trained with aformentioned selected segments. Each class was tested with approx. 30 independent ground control points generated from in-situ registrations (more than 10 control points per class) and visual inspection of the imagery. The high accuracy is due to the limited number of classes, the limited areal coverage, and a significant percentage of the area being covered by user-selected segments.

Class	Affected heath	Fen	Bedrock	Water	Non-affected heath
Affected heath	29	0	0	0	1
Fen	0	29	0	0	1
Bedrock	0	0	28	0	2
Water	1	0	0	28	1
Non-affected heath	3	0	0	0	27
Overall accuracy	0.94				
